# Immunization against a merozoite sheddase promotes multiple invasion of red blood cells and attenuates *Plasmodium* infection in mice

**DOI:** 10.1186/1475-2875-13-313

**Published:** 2014-08-12

**Authors:** Ryan C Smith, Daisy D Colón-López, Jürgen Bosch

**Affiliations:** W Harry Feinstone Department of Molecular Microbiology and Immunology, Johns Hopkins Bloomberg School of Public Health, Baltimore, MD USA; Department of Biochemistry and Molecular Biology, Johns Hopkins Bloomberg School of Public Health, Baltimore, MD USA; The Johns Hopkins Malaria Research Institute, Johns Hopkins Bloomberg School of Public Health, Baltimore, MD USA

**Keywords:** Subtilisin 2, *Plasmodium*, *Anopheles gambiae*, Malaria

## Abstract

**Background:**

Subtilisin-like protease 2 (SUB2) is a conserved serine protease utilized by *Plasmodium* parasites as a surface sheddase required for successful merozoite invasion of host red blood cells and has been implicated in ookinete invasion of the mosquito midgut. To determine if SUB2 is a suitable vaccine target to interfere with malaria parasite development, the effects of SUB2-immunization on the *Plasmodium* life cycle were examined in its vertebrate and invertebrate hosts.

**Methods:**

Swiss Webster mice were immunized with SUB2 peptides conjugated to Keyhole limpet hemocyanin (KLH) or KLH alone, and then challenged with *Plasmodium berghei*. To determine the effects of immunization on parasite development, infected mice were evaluated by blood film and Giemsa staining. In addition, collected immune sera were used to perform passive immunization experiments in non-immunized, *P. berghei*-infected mice to determine the potential role of SUB2 in parasite development in the mosquito.

**Results:**

Following *P. berghei* challenge, SUB2-immunized mice develop a lower parasitaemia and show improved survival when compared to control immunized mice. Moreover, SUB2 immunization results in an increase in the number of multiply invaded red blood cells, suggesting that SUB2 antibodies interfere with merozoite invasion. Passive immunization experiments imply that SUB2 may not have a major role in ookinete invasion, but this requires further investigation.

**Conclusion:**

By interfering with red blood cell invasion, immunization against SUB2 limits malaria parasite development and confers protection from severe malaria. Together, these results provide proof-of-principle evidence for future investigation into the use of SUB2 as a vaccine or drug target to interrupt parasite development in more relevant human malaria models.

**Electronic supplementary material:**

The online version of this article (doi:10.1186/1475-2875-13-313) contains supplementary material, which is available to authorized users.

## Background

Obligate intracellular parasites from the genus *Plasmodium* are the agents responsible for malaria, placing an estimated 3.4 billion people at risk of the disease throughout the world [1]. Five species of *Plasmodium* parasites cause human malaria, yet the largest impacts to public health are primarily caused by *Plasmodium falciparum* in sub-Saharan Africa [2].

Malaria parasites undergo a complex life cycle in their mosquito and human hosts, which require *Plasmodium* parasites to invade and replicate in multiple cell types and host environments. To accomplish these developmental progressions, *Plasmodium* parasites utilize specific invasion ligands and proteases to facilitate host cell invasion [3, 4]. Merozoite invasion of red blood cells (RBCs) has been studied in the most detail and involves a large repertoire of surface proteins that contribute to multiple invasion pathways [3]. Similarly, recent evidence suggests that ookinete invasion of the mosquito midgut may also involve multiple surface proteins and invasion pathways [5]. While both merozoite invasion of the RBC and ookinete invasion of the midgut are rapid, these stages have attracted recent attention as targets for a blood stage [6–8] or transmission-blocking vaccines [9–11].

As a shared component of merozoite and ookinete invasion pathways, subtilisin-like protease 2 (SUB2) is an ideal candidate to interfere with the disease-causing forms of malaria asexual development, as well as development in the obligate mosquito host. In merozoites, SUB2 accumulates in the parasite micronemes and is secreted onto the merozoite surface upon schizont rupture [12]. There, it is believed that SUB2 interacts with an actin-dependent motor to behave as a sheddase, cleaving surface-bound MSP1 and AMA1 on the parasite membrane [12, 13]. As SUB2 moves to the posterior end of the merozoite during RBC invasion, these substrates are cleaved at a certain distance relative to the membrane with minimal sequence specificity, in contrast to other proteases [12]. While little is known regarding SUB2 function during ookinete invasion, limited evidence would suggest that it is secreted by ookinetes during mosquito midgut invasion [14]. In cells that have undergone ookinete invasion, SUB2 is found in protein aggregates in close association with the actin cytoskeleton and may function to disrupt the host cytoskeletal network to facilitate invasion [14]. While evidence would suggest that SUB2 is an integral component of *Plasmodium* parasite development due to its crucial role in RBC invasion [12, 15], attempts to further define its role in the sexual stages of parasite development have yet to be explored.

Although these invasive stages are transient, both stages likely require SUB2 activity for the processing and shedding of parasite surface ligands. Despite the short window of opportunity to target these stages, naturally acquired immunity predominantly targets proteins involved in merozoite invasion [7, 8]. Included among several merozoite surface antigens or proteins secreted during merozoite invasion, SUB2 was determined to be a strong target candidate to elicit malaria protective immunity [7].

To determine if SUB2 is a viable malaria vaccine candidate targeting both the asexual and sexual life cycles of *Plasmodium*, two synthetic peptides were designed representing solvent exposed regions of the *Plasmodium berghei* SUB2 catalytic domain to evaluate the effects of SUB2 immunization in mice. In the present study immunization with peptides directed at *P. berghei* SUB2 confers protective immunity in mice from developing severe malaria infection by attenuating parasite growth via promoting aberrant merozoite invasion. These results therefore validate SUB2 as a novel target against malaria infection in a mouse model system.

## Methods

### SUB2 homology modeling and visualization

Homology model of *P. berghei* SUB2 (PlasmoDB code: PBANKA_091170, Gene ID: 3423789) was generated using the I-TASSER protein structure and function prediction server using default settings [16]. From all the models predicted by the server, the one with the highest confidence score was used for further modeling. Models were visualized using PyMol (PyMoL Molecular Graphics System, Version 1.6.0.0 Schrödinger, LLC).

### Mice

Female Swiss Webster mice (~21-24 g) were purchased from Harlan and maintained in accordance with the recommendations of the Guide for the Care and Use of Laboratory Animals of the National Institutes of Health. All animal procedures were approved by the Institutional Animal Care and Use Committee of the Johns Hopkins University (Protocol number MO09H58).

### SUB2 immunization

Synthetic SUB2 peptides conjugated to keyhole limpet haemocyanin (KLH) through the cysteine at the N- (Sub2 Peptide #2- CRTSIKIVSKDKKTI) or C-terminus (Sub2 Peptide #1- KYSDRYEMTDELFDC) via a –SH bond were produced by GenScript Corporation (Piscataway, NJ, USA).

Female Swiss Webster mice (~21-24 g) were primed with a 50:50 mixture (50 μg/mouse) of both SUB2 peptides in phosphate-buffered saline (PBS) or 50 μg of a control KLH carrier in PBS with either complete Freund’s adjuvant (CFA) or incomplete Freund’s adjuvant (IFA) in a 1:1 emulsion and immunized by intraperitoneal injection (ip). Mice were boosted four times in two week intervals with 50 μg/mouse of peptide in a 1:1 emulsion with IFA via ip injection. Serum was collected from each individual mouse prior to priming, as well as the third and fourth boosting immunizations to monitor antibody titres. Two weeks after the final boosting immunization, animals were used for subsequent challenge experiments with *P. berghei* parasites*.*

### *Plasmodium berghei*and *Plasmodium falciparum*RNA isolation and cDNA production

*Plasmodium berghei* strain ANKA 2.34 total RNA was prepared from blood of an infected Swiss Webster mouse (~10% parasitaemia) obtained via cardiac puncture and isolated using TRIzol Reagent (Invitrogen, Carlsbad, CA) according to the manufacturer’s specifications. Two μg of total RNA was used as a template for the production of cDNA using SuperScriptIII (Invitrogen, Carlsbad, CA).

Approximately 1 μg of total RNA from asynchronized *P. falciparum* strain 3D7 parasites was isolated using TRI Reagent (Molecular Research Center, Inc, Cincinnati, OH) and treated with DNase I (New England Biolabs, Ipswich, MA) according to the manufacturer’s protocol. Synthesis of complementary DNA was performed with the SuperScript First-Strand Synthesis System for RT-PCR (Invitrogen).

### *Plasmodium*SUB2 cloning

*Plasmodium berghei* SUB2 N476 - N1185 (PlasmoDB code: PBANKA_091170, Gene ID: 3423789) and *P. falciparum* SUB2 N528 - S1135 (PlasmoDB code: PF3D7_1136900, Gene ID: 810927) coding sequences were amplified using cDNA obtained from *P. berghei* ANKA 2.34 or *P. falciparum* 3D7 strains using the respective primers *Pb*SUB2_Fwd: 5′ CTCCATG*GCG*AATAATTCAAATGCATTTTTGAGTGTAGAC 3′, *Pb*SUB2_Rev: 5′ ACGGATCCGTTATCATGCTCATATAAATTATATAAAGC 3′, *Pf*SUB2_Fwd: 5′ ATCCATG*GCG*AATAATAAAAAAATTTTGTTAAATGTTGAT 3′ and *Pf*SUB2_Rev: 5′ ACGGATCCACTATCATATTCATACAAATTATATAAGGC 3′. PCR products were amplified using Phusion® High-Fidelity DNA polymerase (New England Biolabs) with an annealing temperature gradient of 52-70°C for 30 sec, followed by extension at 72°C for 2 min.

SUB2 PCR products were inserted in frame using *Nco*I and *Bam*HI restriction sites into a modified pRSF-1b vector (EMD Millipore, Billerica, MA) for expression as a maltose binding protein (MBP)-fusion protein with a C-terminal 6×His tag for purification and detection purposes as previously described [17]. Positive clones were screened using colony PCR with primers described above and insertion sequences were confirmed by sequencing.

### Recombinant protein expression and purification

MBP-SUB2 fusion constructs were transformed into Rosetta 2 (DE3) competent *Escherichia coli* (Novagen) for protein expression. Cells were grown in the presence of 1.5% glucose and 50 μg/ml kanamycin in 500 ml 1X Terrific Broth media until OD_600_ of ~3.0 and induced with a final concentration of 0.5 mM IPTG. Recombinant proteins were expressed overnight at 20°C under vigorous shaking at 250 rpm.

Bacteria were harvested by centrifugation at 2,500 rpm for 30 min at 4°C. Bacterial pellets were resuspended in lysis buffer (25 mM Tris pH 9.0, 100 mM NaCl) and lysis was performed using an Emulsiflex C5 cells disruptor (Avestin Inc., Ottawa, Canada) at 100 MPa. Whole cell lysates were fractionated by centrifugation at 17,000 rpm for 1 hr at 4°C and the supernatant was applied to an open gravity column (BioRad) containing 1 ml of amylose resin (New England Biolabs) for affinity capture of the MBP-tagged fusion protein. Bound protein was washed with lysis buffer and eluted in the presence of 20 mM maltose. Elution samples from the amylose resin purification steps were applied to an affinity column containing Cobalt-Talon resin (Clontech, Mountain View, CA) for secondary purification with the 6×His tag. Bound protein was washed with lysis buffer and eluted with 200 mM imidazole. Elution samples were concentrated using Nanosep Centrifugal Devices (Sigma-Aldrich, St. Louis, MO) with a 10 kDa cut-off.

### Western blots

Approximately 1.7 μg of recombinant *Pb*SUB2 and *Pf*SUB2, and ~3 μg MBP (fusion protein only) were separated on a 12% SDS-PAGE gel. Following electrophoresis, the gel was washed in diH_2_0 for 10 min and equilibrated in 1X transfer buffer (25 mM Tris, 192 mM glycine, 20% methanol, 0.0375% SDS). Proteins were transferred to a PVDF membrane on a semi-dry transfer cell for 2 hrs under constant voltage (25 V). After transfer, the membrane was blocked with 5% milk in 1X TBST for 30 min (250 rpm at 37°C) and washed three times with 1X TBST. Membranes were incubated overnight at 4°C with serum from SUB2- or KLH- immunized mice at a 1:500 dilution in 1X TBST or with a mouse anti-MBP antibody (Upstate–Millipore, #05-912) at a 1:10,000 dilution in 1X TBST. After three washes with 1X TBST, membranes were incubated with an alkaline phosphatase-conjugated goat anti-mouse antibody (1:5,000 dilution in 1X TBST). Detection was carried out using NBT/BCIP alkaline phosphatase substrates (Promega, Madison, WI).

### *Plasmodium*challenge in SUB2 immunized mice

Following immunization with either the CFA or IFA protocols described above, SUB2 or control KLH mice were infected with ~2×10^2^*P. berghei* mCherry [18] asexual parasites via intravenous (iv) injection as previously performed [19]. To monitor parasite growth, thin smears of tail blood were stained with Giemsa and examined under a microscope to determine parasitaemia (% of infected erythrocytes) every day for ten days. Results were combined for KLH- and SUB2-immunized mice using either the IFA or CFA immunization protocols and significance was determined using linear regression analysis. Statistical comparisons of the parasitaemia at day 10 of infected mice were performed using Mann–Whitney analysis.

To determine the effects of immunization on mouse survival following the above *Plasmodium* challenge, the survival of immunized mice was monitored for 40 days following the initial infection. Statistical differences in the survival curves were determined using a Log-rank (Mantel-Cox) test.

### Multiple invasion analysis

Ten days after infection with *P. berghei*, Giemsa-stained thin smears from SUB2 or KLH immunized mice with measurable parasitaemia were analysed by light microscopy. Independent of parasitaemia, approximately 200 infected RBCs were examined per mouse to determine the number of infected RBCs that contained one or more parasites. The percentage of each invasion phenotype was calculated as the number of invasion events, divided by the total number of infected RBCs (iRBCs). Significance was determined using Mann–Whitney.

### Passive immunization experiments

Swiss Webster mice infected with the mCherry strain of *P. berghei* [18] were examined for similar levels of exflagellation three days after inoculation as previously described [9]. Mice with matching infections were anesthetized and used for blood feeding control (pre-KLH) or treatment (pre-SUB2) groups of *Anopheles gambiae* mosquitoes for 15 min. The anesthetized mice were then taken off the cage and passively immunized (iv) with KLH or SUB2 immune sera (final concentration of 2 mg/ml) and allowed to recover for 15 min. The passively immunized mice were then fed to sibling groups of *An. gambiae* mosquitoes for an additional 15 min to measure any effects on parasite development in the mosquito.

Following feeding, mosquitoes were incubated at 19°C to promote *P. berghei* development. Mosquito midguts were dissected seven days post-blood meal (PBM), and oocysts numbers were counted using a compound fluorescence microscope. Oocyst numbers from two independent experiments were pooled and analysed by Kruskal-Wallis with a Dunn’s Multiple Comparison test to determine significance.

## Results

### Structural modelling of *Plasmodium berghei*SUB2 catalytic domain

**Figure 1:**
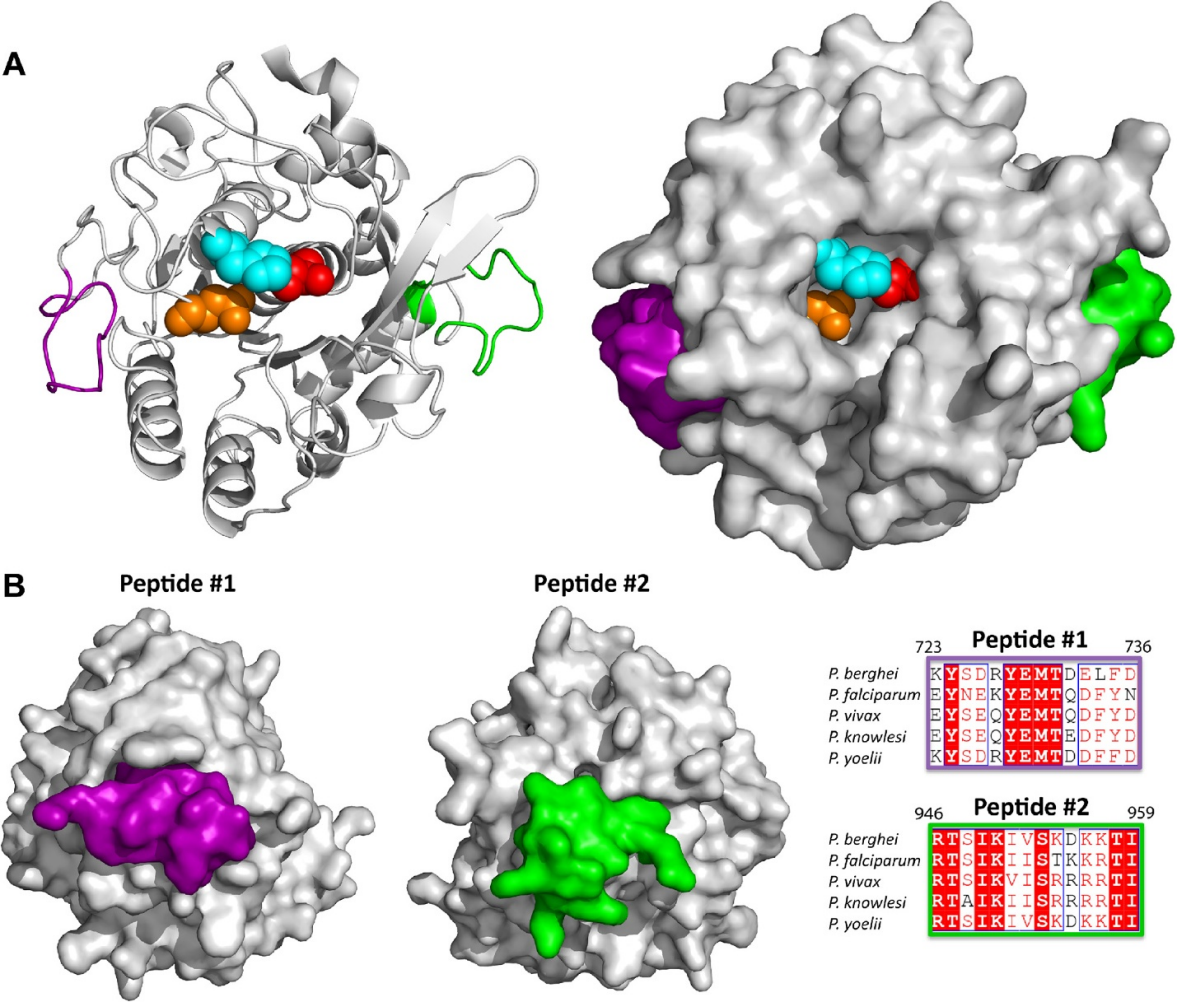
***Pb*** **SUB2 homology models identify peptide targets for immunization. (A)** Cartoon (left) or surface representation (right) homology model of the *Pb*SUB2 catalytic domain (residues L672-L971) visualized with PyMOL software. Regions corresponding to Peptide #1 (purple) and Peptide #2 (green) were used for immunization experiments. Catalytic residues Asp705, His748 and S911 in the active site pocket are shown as orange, cyan and red spheres, respectively. **(B)** Lateral view of Peptide #1 (purple) and Peptide #2 (green) in the *Pb*SUB2 surface representation model reveals that each peptide corresponds to solvent exposed areas (left). Sequence alignments of both peptide sequences with corresponding regions of *P. falciparum*, *P. vivax*, *P. knowlesi,* and *P. yoelii* SUB2 (right). The amino acid position of the first and last residues of each peptide sequence with respect to full length *Pb*SUB2 are shown at the top left and right corner of each alignment. Conserved residues are highlighted with a red background and regions of similarity are marked with red letters against white background. Sequence alignments were visualized using ESPript [21].

A structure model was predicted for the catalytic domain of *Pb*SUB2 by the I-TASSER server and contains a secondary structure topology characteristic of subtilisin-like serine proteases (Figure 1A). The amino acid residues that comprise the catalytic triad Asp 705, His 748 and Ser 911 required for catalysis are positioned at the active site of the model (Figure 1A). Comparing the predicted model using the EBI SSM webserver, the closest structural homologue in the protein data bank (PDB) is the subtilase, thermitase (PDB 1twc:E) from *Thermoactinomyces vulgaris.* With an overall root mean square deviation (RMSD) of 1.4 Å for 247 amino acid residues as determined with PDBeFold [20], therefore the predicted structural model for *Pb*SUB2 therefore has a high confidence level, resembling the overall known fold of other subtilases.

### Design of *Plasmodium berghei*SUB2 peptides

Using proprietary software (GenScript), highly antigenic peptides corresponding to the *Pb*SUB2 catalytic domain were identified. To test these candidate 14 amino acid peptides, the corresponding regions were mapped on a *Pb*SUB2 catalytic domain homology model. Two peptides mapping to opposite flexible solvent exposed regions of *Pb*SUB2 were selected to increase the likelihood that antibodies generated against these peptides would interact with the protease on the surface of merozoites or ookinetes during invasion (Figure 1A). Both peptides correspond to different solvent accessible regions of the *Pb*SUB2 catalytic domain (Figure 1B, left).

The sequence of Peptide #1 is nearly identical (93%) to the corresponding region of *Plasmodium yoelii* SUB2 (Figure 1B, right). The two sequences only differ by the amino acid at position Leu 734 in the *P. berghei* sequence and Phe 734 in *Plasmodium yoelii*, suggesting a high level of conservation between the rodent malaria species. Less conservation exists between Peptide #1 and the human malaria parasites (*P. falciparum*, *Plasmodium vivax* and *Plasmodium knowlesi*), with only 64% similarity (36% ID) to *P. falciparum* (Figure 1B). However, the Peptide #2 sequence alignment reveals more conservation and sequence similarity across *Plasmodium* species. The *P. berghei* and *P. falciparum* SUB2 sequences show 85% similarity (71% ID), while the rodent malaria parasites are completely conserved (Figure 1B). Both peptide sequences map to regions of the *Pb*SUB2 catalytic domain (Figure 1A).

### Mice immunized with SUB2 peptides recognize recombinant *Pb*SUB2

**Figure 2:**
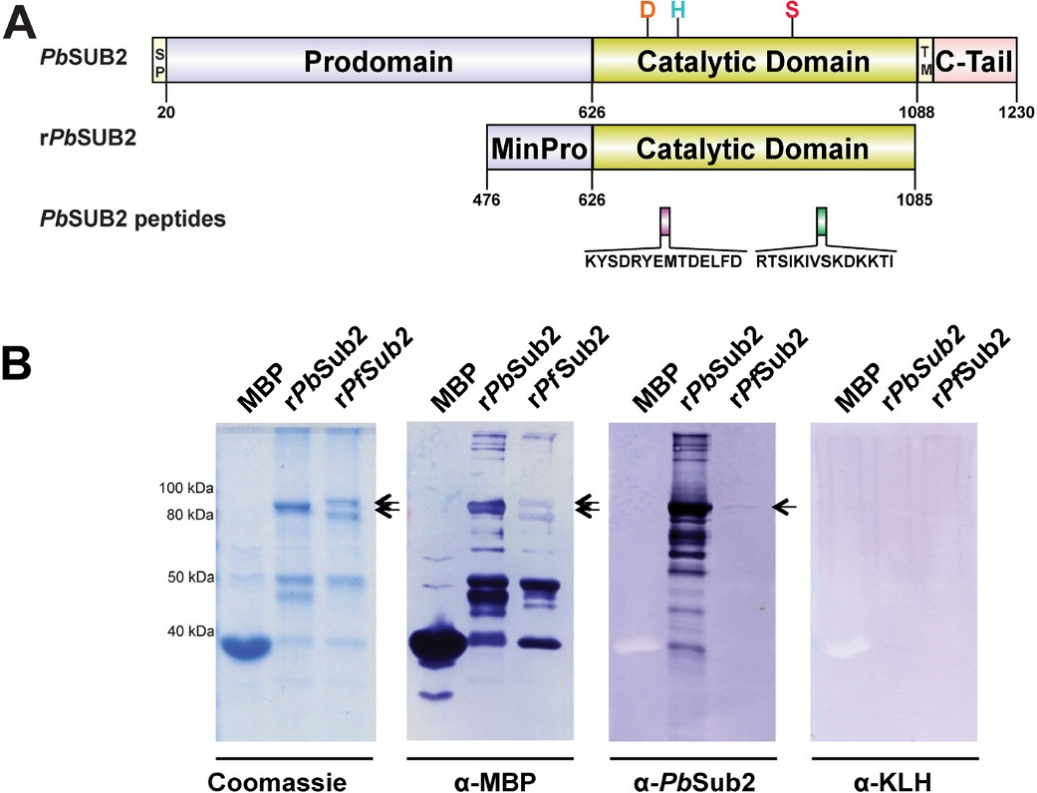
**Production of recombinant SUB2 and recognition using Sub2 immune sera. (A)** Domains of endogenous *Pb*SUB2 (top): signal peptide (residues 1–20), pro-domain (residues 21–626), catalytic domain (residues 627–1,088) with catalytic residues Asp (orange), His (cyan) and Ser (red), transmembrane domain (residues 1,089–1,111) and cytoplasmic tail (residues 1,112–1,230). Representation of recombinant *Pb*SUB2 (middle) containing a minimal inhibitory domain and the full catalytic domain. Below, *Pb*SUB2 Peptides #1 (purple) and #2 (green) are aligned to endogenous *Pb*SUB2 and r*Pb*SUB2 with peptide sequences. Illustrations were prepared with DOG1.0 [22]. **(B)** Recombinant proteins maltose binding protein (MBP), *Pb*SUB2 or *Pf*SUB2 MBP-fusion proteins were separated on polyacrylamide gels and stained with Coomassie, or transferred and visualized by Western Blot with specific MBP, SUB2, or KLH antibodies. Arrows denote full length *Pb*SUB2 and *Pf*SUB2 recombinant products. Approximate sizes in kilodaltons (kDa) are displayed on the left.

MBP-SUB2 expression constructs containing a short region of the pro-domain and the entirety of the SUB2 catalytic domain (Figure 2A) were expressed using a Rosetta2 *E. coli* heterologous system. Recombinant SUB2 was visualized as a single band for *Pb*SUB2, or as two bands for *Pf*SUB2, of approximately 110 kDa full-length protein products (Figure 2B). Smaller protein products are likely the result of either sample degradation during the purification process or translational truncation products that were observed for both SUB2 constructs (Figure 2B). The truncation products can be explained by the occurrence of numerous rare-codons within the SUB2 gene, leading to premature termination during translation. Both full-length and truncated forms of SUB2 were detected using an MBP antibody, confirming the detection of the recombinant MBP-SUB2 fusion protein products (Figure 2B). When incubated with immune sera from SUB2-immunized mice, recombinant *Pb*SUB2 is detected in full length and degraded forms while only a faint band corresponding to full-length recombinant *Pf*SUB2 protein was detected (Figure 2B). Importantly, mice immunized with KLH alone did not recognize either recombinant SUB2 protein (Figure 2B).

These results confirm that antibodies were generated in mice immunized with *Pb*SUB2 peptides that can sufficiently recognize recombinant *Pb*SUB2 (Figure 2B). Furthermore, immune sera raised against *Pb*SUB2 peptides specifically targets *Pb*SUB2 with minimal cross-reactivity to *P. falciparum* SUB2 (Figure 2B), suggesting that the observed conservation in the peptide sequences is inadequate for cross-species protection. However, future immunization experiments are needed to determine the properties of the individual peptides and whether they are capable of cross-species immune recognition of different *Plasmodium* species.

### SUB2-immunization impairs asexual *Plasmodium*development

**Figure 3:**
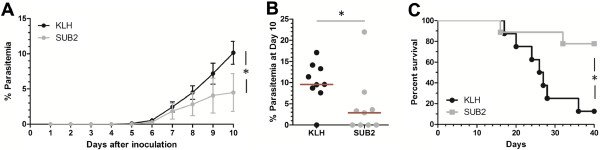
**SUB2 immunization reduces the intensity of** ***Plasmodium berghei*** **infection and increases mouse survival.** The parasitaemia of KLH- or SUB2-immunized mice was determined over the period of ten days after infection with *P. berghei* parasites **(A)**. Each point represents the mean parasitaemia (n = 9) with error bars displaying standard errors of the mean and the asterisk denoting significance (*P* = 0.0042). The scatter plot displays the parasitaemia at day 10, with each point representing the parasitaemia of individual KLH- or SUB2-immunized mice **(B)**. The red bar represents the median of each experiment with the asterisk denoting significance (*P* < 0.05). The survival of KLH- and SUB2-immunized mice was monitored over the course of forty days following *P. berghei* challenge **(C)**. The number of surviving mice for each treatment over the duration of the experiment is displayed as a percentage of the total number of infected mice at a given time point. Statistical differences are marked by an asterisk (*P* = 0.0082).

To monitor the effects of immunization on parasite development, KLH- and SUB2-immunized (IFA or CFA) mice were challenged with ~2×10^2^*P. berghei* parasites by intravenous injection and the parasitaemia was monitored over the period of ten days. Blood stage infections were detected in 17 of 18 mice, and little variation was seen between mice immunized with the IFA or CFA immunization protocols (Table 1). As a result, both immunization experiments were pooled for analysis and are summarized in Table 1. Compared to control KLH-immunized mice, SUB2-immunized mice showed a slight, but not significant delay in the pre-patency of infection (Table 1). However, when the parasitaemia was monitored over the period of ten days (Additional file 1), asexual growth was significantly reduced and in some mice completely attenuated following SUB2-immunization (Figure 3A).

**Table 1:** **Summary of immunization experiments**

Experiment	Adjuvant	Antigen	# Mice	Infected	Pre-patency	Clearance*	Mean survival ^§^
1	IFA	KLH	6	5/6	6.2	0/5	27.3
		SUB2	6	6/6	6.7	4/6	34.6
2	CFA	KLH	3	3/3	6	0/3	31.3
		SUB2	3	3/3	6.7	0/3	40+
*Total*		*KLH*	*9*	*8/9*	*6.1*	*0/9*	*28.6*
		*SUB2*	*9*	*9/9*	*6.7*	*4/9*	*36.4*

*Mice with detected parasitemia that had cleared the parasite infection (measured at Day 10).

^§^Average number of days mice survived following *P. berghei* challenge.

In SUB2-immunized mice, parasite growth was reduced by 37, 43, and 56% from days 8–10, effectively reducing parasitaemia more than two-fold when compared to KLH control mice (Figure 3A). In addition, nearly half of the SUB2-immunized mice (four of nine) had cleared all signs of parasite infection by day 10 (Figure 3B and Table 1). None of the KLH-immunized mice infected with *P. berghei* was able to clear the infection over the duration of the experiment (Figure 3B and Table 1).

### SUB2-immunization increases mouse survival after *Plasmodium berghei*challenge

Since *P. berghei* asexual development is reduced in SUB2-immunized mice (Figure 3A), the possibility that SUB2-immunization also protects mice against malaria lethality through the decreased parasite burden was further explored.

After initially monitored for parasitaemia (Figure 3A), the same KLH- and SUB2-immunized mice were monitored for a total of 40 days to examine survival following *P. berghei* challenge (Figure 3C). In these experiments, SUB2-immunized mice showed increased survival over control KLH-immunized mice (Figure 3C and Table 1). On average, SUB2-immunized mice survived for more than one week longer than KLH control mice (Table 1), and seven of nine mice survived the duration of the experiment (Figure 3C). In contrast, only one of the eight infected KLH mice survived the entire 40-day period (Figure 3C). This would suggest that the attenuated malaria parasite growth seen in SUB2-immunized mice (Figure 3A) also translates to an increased survival following *P. berghei* challenge (Figure 3C).

### SUB2-immunization promotes aberrant red blood cell invasion

**Figure 4:**
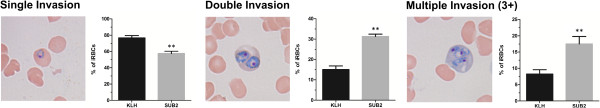
**SUB2-immunization promotes multiple invasion of red blood cells.** Representative images of single, double, or multiple invasion (3+) events in *P. berghei-*infected red blood cells are depicted with their corresponding percentages in KLH- (black) or SUB2- (grey) immunized mice at ten days post-infection. The percentage of each invasion phenotype is displayed as the mean and standard error. Asterisks denote significant differences between KLH-and SUB2-immunized mice (*P* < 0.01).

Based upon observations measuring the parasitaemia of the immunized mice (Figure 3A), there appeared to be a noticeable increase in the number of infected RBCs with multiple parasites in SUB2-immunized mice. To quantify these presumed defects in invasion, the percentages of infected RBCs that had one, two, or multiple (three+) parasites were measured in KLH- and SUB2-immunized mice (Figure 4). Validating previous observations, SUB2-immunized mice had a significant decrease in the number of infected RBCs that had undergone a single invasion event when compared to KLH-control mice (Figure 4). In turn, corresponding increases in the number of double or multiple invasion events (three+) following SUB2 immunization were also detected (Figure 4).

Based upon these data and the important functional role of SUB2 in RBC invasion [12, 15], it is clear that SUB2-immunization interferes with merozoite invasion. Although it is not completely understood how SUB2-immunization might influence the production of these aberrant invasion events, previous studies using antibodies to merozoite surface proteins similarly report phenotypes promoting multiple invasion [23, 24].

### SUB2 immune sera does not interfere with ookinete invasion in passively immunized mice

One previous study has reported that SUB2 is expressed by ookinetes, implicating that SUB2 may be secreted into the cytoplasm of ookinete-invaded cells as the parasite traverses the midgut epithelium [10]. Immunofluorescence staining identified SUB2 protein aggregates in close proximity to the actin cytoskeleton, which suggests SUB2 may play an important role in cytoskeleton modifications during the process of ookinete invasion [14].

**Figure 5:**
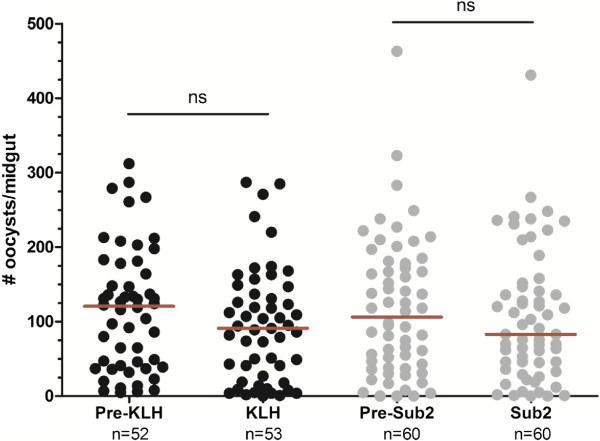
**Passive immunization with SUB2 immune sera does not influence parasite growth in the mosquito.** Oocyst numbers were measured to determine the effects of passive immunization to control KLH- or SUB2-immune sera. *Plasmodium berghei*-infected mice were fed to mosquitoes and oocyst numbers were determined for each experimental group before passive immunization (pre-KLH or pre-SUB2), or following passive immunization (KLH or SUB2). The total number (n) of mosquito midguts examined is displayed under each experimental group. The red bar denotes the median of each experiment. No significant (ns) differences were identified for either experimental group following passive immunization.

To address the role of SUB2 in ookinete midgut invasion and the potential role that SUB2 immune sera could also inhibit ookinete invasion, passive immunization assays were performed to determine the effects on parasite development in the mosquito. As expected, passive immunization with the control KLH immune sera did not significantly alter *Plasmodium* oocyst numbers (Figure 5). Similarly, passive immunization with SUB2 immune sera did not significantly alter oocyst numbers (Figure 5), suggesting that SUB2 may either not be required for ookinete invasion of the mosquito midgut or that the immune sera was present in suboptimal levels needed to inhibit ookinete invasion. These research questions highlight the need for further investigation into the role of SUB2 during the mosquito stages of *Plasmodium* development.

## Discussion

Although more than 40% of the world’s population is at risk of malaria transmission, only limited resources exist to readily combat *Plasmodium* parasites. Current drug therapy faces the ever-increasing risk of resistance [25] and while multiple approaches have thus far been employed to create a malaria vaccine, they have had only mixed results in clinical trials [26]. As a result, new strategies to reduce malaria transmission are desperately needed.

*Plasmodium* species utilize many different proteases during their complex life cycle in the human and mosquito hosts, and serve as optimal targets to interfere with malaria transmission. Previous reports have demonstrated the required role of a *Plasmodium* subtilase (SUB2) for asexual development through its role as a sheddase required for merozoite invasion [12, 15]. Additional studies have also implicated SUB2 in ookinete invasion [14], thus making SUB2 an attractive target to interfere with parasite development in both its human and mosquito hosts.

Using a rodent model, the potential of targeting SUB2 by immunizing mice against specific SUB2-derived peptides was addressed. When compared to control KLH-immunized mice, SUB2-immunization resulted in a slight delay in prepatency, decreased parasitaemia when monitored over a ten-day period, and increased survival following infection. Similar results were obtained independent of the method of immunization, suggesting that the effects of immunization are primarily that of the SUB2 antigens and not from non-specific effects mediated by the CFA. Together, these data would suggest that SUB2-immunization greatly impairs parasite growth, likely by interfering with the efficacy of merozoite invasion.

In support of this idea, an increase in the number of multiple-invaded RBCs following SUB2-immunization was detected, suggesting that merozoite invasion is significantly altered. Similar effects have been seen in other studies using antibodies targeting merozoite proteins, where it was proposed that multiple invasions are the result of merozoite agglutination [23, 24]. According to this hypothesis, the invasion of some merozoites may be completely blocked, while incomplete inhibition may result in multiple parasites that have been cross-linked by SUB2 antibodies that undergo invasion together as a complex or dissociate once the RBC surface has been recognized. Alternatively, if RBC invasion is slowed in the presence of SUB2 antibodies, multiple merozoites may invade the same cell before the parasite can direct modifications to the RBC surface to prevent further invasion. Due to the short time frame in which merozoites undergo release and invasion into new RBCs, the concentration and rate of antibody binding may be critical factors in invasion inhibition.

Very little information exists regarding the viability of infected RBCs that have undergone multiple invasion events. It has been hypothesized that nutritional and structural limitations following multiple invasion may reduce the production of viable merozoites [24], thus raising the possibility that these infected RBCs may be a ‘dead-end’ for the parasite. As a result, the higher incidence of multiple invasions may have a significant contribution to the decreased parasitaemia and increased survival in the SUB2-immunized mice.

While the increased survival of SUB2-immunized mice would suggest that SUB2 immunization can confer protection to the severe forms of malaria infection, the potential reasons for morbidity in the *Plasmodium*-infected mice was not further explored. Visible neurological symptoms of cerebral malaria (as defined by [27]) were not observed, suggesting that infection-induced mortality was due to other malaria-related causes.

Based upon previous studies implicating SUB2 in ookinete invasion [14], the role of SUB2 on parasite development in the mosquito was examined using passive immunization assays. Although differences in oocyst development were not detected, it still remains unclear what role SUB2 may have during the process of ookinete invasion. Given the limited amount of immune sera produced, only one concentration was tested in the passive immunization experiments and these may have been suboptimal concentrations to inhibit ookinete invasion. Alternatively, the production of SUB2 by ookinetes may not be integral to ookinete motility within the mosquito midgut and may not be a viable target to interfere with malaria transmission. As a result, the role of SUB2 in *Plasmodium* ookinetes requires future study.

## Conclusion

These experiments indicate that immunization against a merozoite sheddase can interfere with *Plasmodium* development in mice. While the results are still preliminary using a rodent malaria model, the data provide strong evidence for future investigation into the use of SUB2 as a vaccine or drug target to interrupt parasite development in more relevant human malaria models. In support of this idea, epidemiological studies in Papua New Guinea indicate a strong correlation between the detection of SUB2 antibodies and naturally acquired protective immunity [7]. Similar studies with blood samples from field isolates of African populations to determine the role of SUB2 in naturally acquired immunity could provide further verification for this promising approach as a vaccine candidate. As a result, future experiments will address challenges to increase the efficacy to inhibit SUB2 function using monoclonal antibodies or small molecules inhibitors to interrupt merozoite invasion.

## Electronic supplementary material

Additional file 1: **Parasitaemia of individual immunized mice following** ***Plasmodium berghei*** **challenge.** (PDF 230 KB)
