# Supplementary material for: Immunization against a merozoite sheddase promotes multiple invasion of red blood cells and attenuates Plasmodium infection in mice
Source: Malar J. 2014 Aug 12;13:313. doi: 10.1186/1475-2875-13-313 (PMC4248431; doi:10.1186/1475-2875-13-313)
Supplement: Supplementary file 1 — Additional file 1: Parasitaemia of individual immunized mice following Plasmodium berghei challenge. (PDF 230 KB) [file 12936_2014_3348_MOESM1_ESM.pdf]

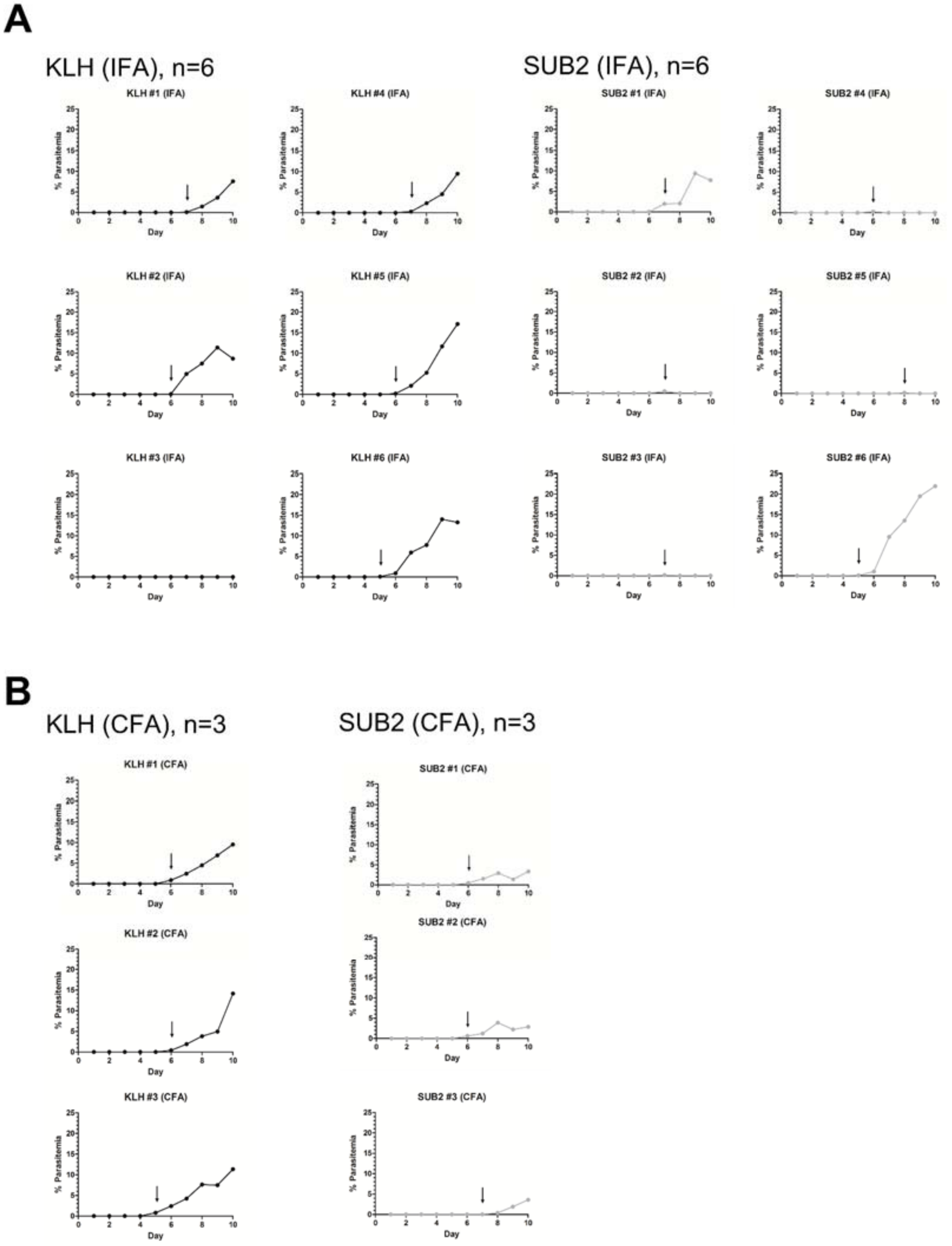


**Additional file 1: Parasitemia of individual immunized mice following *P. berghei* challenge.** The parasitemia of KLH- or SUB2-immunized mice was determined over the period of ten days after infection with $2x10^2$ *P. berghei* parasites. Results are shown for individual KLH- and SUB2-immunized mice using the IFA (n=6) (A), or CFA (n=3) (B) immunization protocols. Each point represents the parasitemia at a given time point and the arrow depicts the day that parasites were first detected (pre-patency).
